# Future Perspectives of Proton Therapy in Minimizing the Toxicity of Breast Cancer Radiotherapy

**DOI:** 10.3390/jpm11050410

**Published:** 2021-05-13

**Authors:** Marika Musielak, Wiktoria M. Suchorska, Magdalena Fundowicz, Piotr Milecki, Julian Malicki

**Affiliations:** 1 Electro-Radiology Department, Poznan University of Medical Sciences, 61-701 Poznan, Poland; wiktoria.suchorska@wco.pl (W.M.S.); piotr.milecki@wco.pl (P.M.); julian.malicki@wco.pl (J.M.); 2 Greater Poland Cancer Centre, Radiobiology Laboratory, Department of Medical Physics, 61-866 Poznan, Poland; 3 Greater Poland Cancer Centre, Radiotherapy Ward I, 61-866 Poznan, Poland; magdalena.fundowicz@wco.pl; 4 Greater Poland Cancer Centre, Medical Physics Department, 61-866 Poznan, Poland

**Keywords:** proton therapy, cardiotoxicity, breast cancer, radiotherapy

## Abstract

The toxicity of radiotherapy is a key issue when analyzing the eligibility criteria for patients with breast cancer. In order to obtain better results, proton therapy is proposed because of the more favorable distribution of the dose in the patient’s body compared with photon radiotherapy. Scientific groups have conducted extensive research into the improved efficacy and lower toxicity of proton therapy for breast cancer. Unfortunately, there is no complete insight into the potential reasons and prospects for avoiding undesirable results. Cardiotoxicity is considered challenging; however, researchers have not presented any realistic prospects for preventing them. We compared the clinical evidence collected over the last 20 years, providing the rationale for the consideration of proton therapy as an effective solution to reduce cardiotoxicity. We analyzed the parameters of the dose distribution (mean dose, Dmax, V5, and V20) in organs at risk, such as the heart, blood vessels, and lungs, using the following two irradiation techniques: whole breast irradiation and accelerated partial breast irradiation. Moreover, we presented the possible causes of side effects, taking into account biological and technical issues. Finally, we collected potential improvements in higher quality predictions of toxic cardiac effects, like biomarkers, and model-based approaches to give the full background of this complex issue.

## 1. Introduction

Proton radiotherapy is the state-of-the-art approach in cancer treatment and it is usually proposed as a better alternative to photon radiotherapy. Because of the more favorable dose distribution in the target volume, many studies indicate that proton therapy should be prescribed for cancers such as chondrosarcomas, chordomas of the skull base, ocular tumors, and pediatric cancers [1]. A lot of ongoing clinical trials could show the benefit of proton over photon therapy in such primary tumor locations such as head and neck, prostate, gastrointestinal tract, lung, and central nervous system tumors, mainly reirradiated after additional primary definitive photon therapy. Radiation therapy with protons originated in 1946, when Wilson first proposed using protons in radiotherapy because of the more favorable dose distribution [2,3]. The first place that started treating patients was Loma Linda University in 1990, with a synchrotron that accelerated protons to 250 MeV [4]. There are currently 109 proton-based treatment centers globally, of which 37 are located in the United States, based on the information from The Particle Therapy Co-Operative Group updated in September 2020 [5].

**Figure 1:**
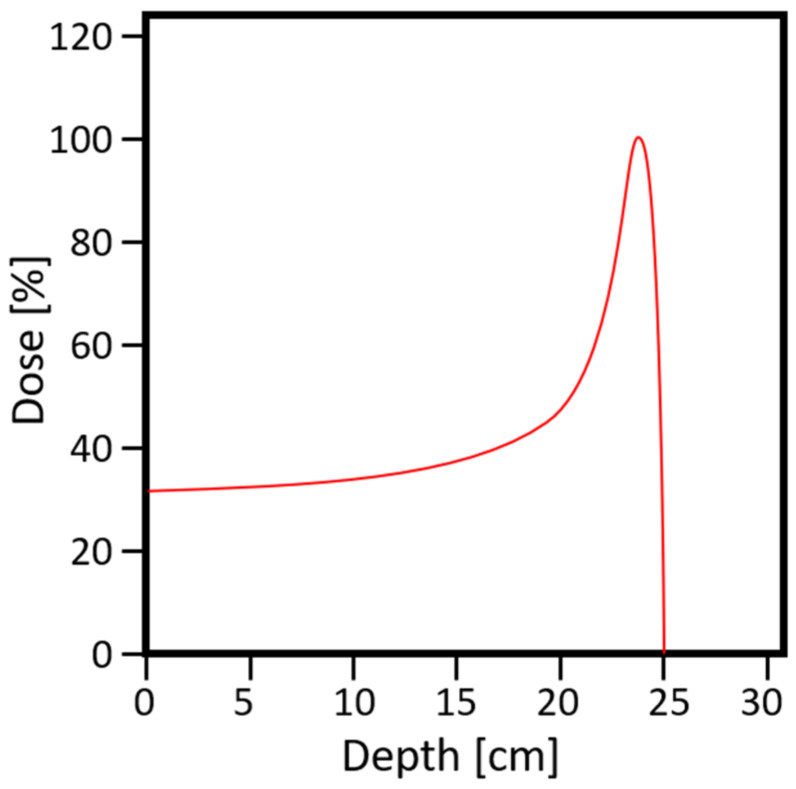
Characteristic dose distribution of the proton beam.

Proton therapy is perceived as a beneficial and efficient method of cancer therapy, but it is also associated with certain limitations [6]. One of the critical topics discussed regarding the competition between photon and proton radiation are the effectiveness treatment, dose distribution in organs at risk (OAR), and total integral dose in normal tissue [7]. The main difference is in the interaction with the tissues of protons and photons. The dose transferred in the medium decreases exponentially for photons, while for protons, the depth distribution of the dose has a characteristic shape, called a Bragg peak (Figure 1). For proton radiation, the maximum dose occurs at a wide range of depth, depending on the beam energy. Because of the high dose gradient, it is possible to deliver radiation to the tumor volume while avoiding excessive radiation of the surrounding tissues [8]. For this reason, a lower probability of complications in healthy tissues can be expected. This provides the opportunity for improvement in the local control of cancer. Proton therapy centers are more diverse in terms of technological and functional solutions than conventional radiotherapy centers. Each institution has a beam with slightly different parameters, and there are also unique ways of immobilizing a patient or administering a dose.

The current priority in radiotherapeutic treatment is the highest possible beam conformality and full coverage of the irradiated volume while maintaining healthy tissues around the irradiated area without any damage [9]. Currently, proton therapy is not considered as the standard care for patients with breast cancer [10]. A possible cause of this may be the knowledge gap in clinical evidence, as there is little research confirming the advantage of protons over photons [11].

To properly assess the advantages and effectiveness of proton therapy, investigators should take into account factors such as dose values in OARs like the heart, lungs, the left anterior descending artery (LAD); irradiation techniques; the number of used fields; breast size and volume; molecular and microenvironmental factors of the tumor; hormone-dependence; radiosensitivity of tumor cells; treatment combined with surgery or chemotherapy; possible skin toxicity; age; and, finally, the patient’s preference [12,13]. There are various technics like conventional whole-breast irradiation (WBI), partial breast irradiation with or without nodal irradiation, post-mastectomy chest wall radiotherapy, and accelerated partial breast irradiation (APBI). It is crucial to evaluate the cardiac dose sparing technique in order to minimize the side effects of radiotherapy. Structures like the heart, including the pericardium, myocardium, valves, conduction system, and coronary arteries have the potential to be at risk during exposure to irradiation [14]. Some analyses have confirmed a wide range of different side effects from other organs at risk, such as cardiopulmonary toxicity, rib fractures, fat necrosis, dermatitis, radiation pneumonitis, or even cardiac death after irradiation. Furthermore, it is essential to assess patients subsets that may have a specified benefit from protons. To achieve individual prediction of later cardiovascular effects, it is necessary to create a precise individual risk estimation, including every crucial treatment [15].

This review aims to analyze and evaluate the latest literature reports on breast cancer treatment with the use of proton beam irradiation. We aim to compare the clinical evidence collected over the last 20 years to provides the basis for proton therapy’s valuable consideration as an effective solution to reduce cardiotoxicity. Moreover, possible causes of undesirable effects will be presented, taking into account biological and technical issues. Finally, we would like to indicate potential improvements in higher quality predictions of toxic cardiac effects, like biomarkers and model-based approaches.

## 2. Review Methodology

The literature search was conducted for papers related to breast cancer proton therapy. Moreover, the search included papers dealing with cardiotoxicity and skin damage as the main examples of proton radiotherapy side effects. The publications were searched for using the PubMed database, including papers published from 2002 to 2020.

## 3. Toxicity of Breast Cancer Proton Therapy

### 3.1. Cardiopulmonary Toxicity

The organs most exposed to radiation during radiotherapeutic treatment are the heart, in particular the coronary arteries, the left anterior descending coronary artery (LAD), the pericardium, the myocardium, the valves, the conduction system; lungs; and the chest wall and skin [16]. Moreover, investigators have shown the effect of calcification of the mural endocardium and the large arterial intima, which is associated with the previous radiation treatment [17].

There have been many studies [11,18,19,20] confirming the cardiotoxicity of radiotherapy in breast cancer. The most frequently cited work is the analysis done by Darby et al. [14]. They performed a population-based case-control study of major coronary events in 2168 women, of whom 963 suffered from major coronary events. The group focused on evaluating the mean radiation doses to the heart and LAD. The main aim of the study was to estimate the impact of radiation treatment on the incidence of heart disease in patients assessing the cardiac risk factors and radiation-related diseases. The study results revealed that incidental cardiac exposure during the irradiation of breast tumors increased the risk rate for major coronary events by 7.4% per Gray. This work is a stimulus to broaden the knowledge about cardiac diseases, specify the criteria for qualifying patients for radiotherapy, and improve the quality of treatment using modern technological solutions. The most common radiation-related side effects are rib fractures, the coronary artery blockage, interstitial fibrosis, valvular abnormalities, myocardial dysfunction, pericardial disease, congestive heart failure, cardiomyopathies, arrhythmias, and conductive disturbances [21]. Radiation-induced heart disease (RIHD) is the focus direction because of the still unknown dose-related parameters, crucial in risk assessment [19]. Moreover, a lack of knowledge about the patterns or pathways of cardiac structure damage should be fulfilled by in vitro and in vivo studies [22].

All individual patient factors should be taken into account when assessing the risk of side effects. One of such factors is the presence of preexisting cardiac disease, which is a potential radiotherapy cardiac risk factor. The only way to avoid heart disease is to avoid radiation therapy, but studies show that radiation therapy increases survival in breast cancer patients. In order to avoid the undesirable effects caused by preexisting cardiac risk factors, it is necessary to meticulously identify and monitor cancer patients [23]. The European Society of Medical Oncology has presented guidelines for the prevention, diagnosis, and management of cardiovascular disease associated with cancer therapy [24]. This tool highlights the prevention of heart disease through weight loss, exercise, and blood pressure control. Moreover, cardiovascular screening for risk factors and relevant clinical examination should be performed in all patients. Simple methods have been proposed, which are always presented in these types of guidelines, as follows: when qualifying a patient, preexisting risk factors for cardiac adverse events and the need for appropriate cardiac monitoring during and after radiotherapy should be assessed and established. It is necessary to identify patients with asymptomatic cardiac dysfunction, so that breast cancer therapy can be changed and cardiac medication can be implemented [24].

#### 3.1.1. Accelerated Partial Breast Irradiation

Since proton therapy was introduced into widespread use in radiotherapy, its advantages over conventionally used methods have been debated in terms of dose distribution, cost, availability, and results over the years. The main parameters analysed are the coefficient factors describing the conformality of the dose distribution in the target region [25]. One of the commonly used techniques in radiotherapy is accelerated partial breast irradiation. Taghian et al. [26] performed a radiation dose distribution and costs analysis for three-dimensional, conformal beam APBI comparing proton and traditional photon whole-breast irradiation. The data were obtained from a sample of 25 patients treated between 2004 and 2005. They observed that proton techniques exhibited improved target dose conformation, considering reductions in non-target volumes of breast tissue. Moreover, a significant difference was observed in lung doses between protons and photons using the partial breast irradiation technique. The main disadvantages that should be considered are availability and cost of proton radiotherapy. Although it offers a promising prospect in improved clinical and cosmetic follow-up, it should be further monitored and developed. A similar work was carried out by another research group [27]. They aimed to compare the APBI technique using two different kinds of radiation beams, analysing the stage I breast cancer group of 24 patients. They showed that both types of radiation were characterised by a high target coverage.

The effects of various radiotherapy techniques were also investigated in 14 patients diagnosed with deep-seated early-stage breast cancer boosting the tumor bed [28]. Dynamic arc or rapid arc technics were the greatest for such cases, although proton therapy had almost zero OAR doses. The techniques mentioned above were good alternatives for patients treated with APBI. Another work [29] compared two proton beam techniques, namely: the passive scattering proton beam radiotherapy technique and the intensity-modulated proton radiotherapy technique (IMPT). The authors demonstrated that the IMPT plans were significantly superior to breast skin-sparing and normal tissue sparing, although numerical data limiting the robustness of IMPT were also presented. The passive technique for APBI was the most beneficial treatment option in this study. Galland-Girodet et al. [30] conducted a long-term outcome trial based on a seven-year follow-up comparing the proton and photon beams in the 3DCRT technique for APBI. They analysed a group of 98 stage I breast cancer patients, 19 of whom were treated with PBT and 79 of whom were treated with photons or a combination of photons and electrons. They observed that the toxicity of the proton radiation was higher in the presence of such endpoints as telangiectasia (69% of patients), pigmentation changes (54%), and other late skin toxicities (62%). This work was is valuable approach to proton therapy, as lower cardiac and lung dose values were observed, which is an essential criterion for patients with peri-cardiac disease. However, it also had the disadvantage of actual skin toxicity occurring over the years observed. Other scientists showed a dosimetry comparison of results for catheter-based brachytherapy (BT), IMPT, and PBT techniques based on generated plans for 12 left-sided breast cancer patients with a strut-adjusted volume implant (SAVI) [31]. All techniques achieved a comparably similar dose conformality. This study also confirmed the better use of protons in radiotherapy due to dosimetry doses, and emphasised the need to collect further clinical evidence for early-stage breast cancer. The dosimetry dose distribution from all studies is included in Table 1.

#### 3.1.2. Whole Breast Irradiation

One of the most frequently used techniques in patients with breast tumors is whole breast irradiation. It consists mainly of administering a total dose of 50 Gy in about 2 Gy fractions. WBI is a challenge for planners and clinicians because it can be associated with many side effects after radiotherapy. Many different radiation delivery strategies have been tested, depending on geometric factors. The most common case is post-mastectomy, where the target is in the chest wall and nodal areas, or if the patient has undergone breast reconstruction. However, there is still a lack of experimental data on relationship between the tumor’s molecular profile and its response to radiation.

Fogliata et al. [32] analyzed various irradiation techniques considering the conventional photon beam, IMPT, and PBS for difficult geometry patients. They were characterized by a highly concave breast tissue volume around the lung where there was a high risk of lung irradiation. The two-field conventional photon radiotherapy, three-field non-IMRT, two-field IMRT, three-field IMRT, and single-field proton therapy were checked. It was found that using more fields resulted in a significant reduction in the undesirable dose of about 10 Gy. The highest conformality and healthy tissue sparing were achieved with the use of a proton beam. However, because of its quality and possibilities, the three-field non-IMRT technique was introduced into everyday use in difficult breast tumor localization. Another group [33] compared the radiation techniques with photons, IMRT, and protons on breast cancer patients.

The assigned dose was 50 Gy, and the target included breast, internal mammary, supraclavicular, and axillary nodes. In this work, it was observed that IMRT and proton designs had similar results and were comparable with the proton beam’s predominance in health tissue sparing. It was noted that an attempt to reduce the doses of neighboring critical structures in IMRT resulted in a deterioration of the homogeneity of the target coverage. The opposite effect was observed in two-field proton therapy, where a reduction of OAR doses had no effect on conformality with tumor irradiation. The work by Ares et al. [34] is one of the most cited studies when comparing radiotherapeutic techniques. They reviewed various treatment plans for 20 left-sided breast cancer patients using 3D-CRT, IMRT, and IMPT plans. The irradiated target’s complexity characterized their work, as they considered various combinations of the whole breast, chest wall, medial-supraclavicular, lateral-supraclavicular, level III axillary nodes, and internal mammary chain. The aim was to identify the patients for whom proton radiotherapy could be dedicated as the most beneficial treatment strategy in terms of individual patient physiology. The most significant benefits were obtained with IMPT, with both an improved PTV conformality and reduced doses in OAR. It is a promising approach to minimize the side effects after radiotherapy, especially as it increases the nodal sites numbers. They showed that proton therapy positively impacts complex target volumes in patients with pulmonary or cardiac toxicity profiles. Jimenez et al. [35] conducted another work on post-mastectomy radiation in women with bilateral implants. Such cases cause technical difficulties during patient selection and treatment planning. This study aimed to compare IMPT with 3DCRT after bilateral mastectomy and reconstruction in a group of five patients. In this analysis, an improved homogeneity in the target coverage was also observed, considering the chest wall and regional lymphatics. Moreover, it was found that IMPT enables women to undergo radiotherapy after mastectomy without the need for delays in breast reconstruction, which is essential for the patient’s quality of life.

Scientists [36] also wanted to evaluate the potential advantages by comparing the 3D conformal photon and proton radiotherapy with 3DCRT, including photon–electron and IMRT. For this purpose, treatment plans for 10 patients were generated. The analysis showed similar results as in the works described above. The typically assessed criteria take into account conformality and homogeneity in target coverage and low doses in critical organs. In this study, these two parameters were the most appropriate when using proton therapy compared with the other techniques. Fleimer et al. [37] checked one of the most frequently used proton radiotherapy techniques, scanned proton beams. They showed that it could be used to treat multifocal or lobular disease, as, in these cases, cold spots in the PTV usually appear. This implementation also significantly influences the individualization of radiotherapy treatment, as further evidence has shown that it will allow for the proton radiotherapy of patients with geometrically tricky targets. Fagundes et al. [38] carried out a work comparing proton therapy techniques with 3DCRT, helical tomotherapy, and volumetric modulated arc therapy (VMAT). They included data from scans of 10 patients with left-sided stage III breast cancer after a radical mastectomy. The targets were defined as the chest wall, axilla levels I to III, and the supraclavicular and internal mammary nodes (IMN). The study showed that proton plans had the lowest doses in the lungs or healthy breast.

Moreover, proton beam plans were distinguished by their ability to cover IMNs, which could not be achieved using a photon beam without reducing the other parameters’ qualities. The proton beam could be a potential tool to reduce the risk of a second malignancy. Other scientists [39] examined the dose-comparison in OARs like the heart, LAD, and lungs between the proton and the photon beams. In this study, treatment sessions were conducted with the deep inspiration breath holding (DIBH) technique at IMRT. New plans were re-generated by implementing proton beam radiation with uniform scanning (US) and pencil beam scanning (PBS) techniques. The target coverage was comparable between the photons and protons; however, using proton radiation had much lower doses in the OARs. The influence of proton therapy (PT) on regional nodal irradiation (RNI) was also checked by comparing the dosimetry values between PT and conventional therapy [40]. Over the four years, 18 patients requiring RNI were included in this analysis. Proton therapy showed better results for all of the patients, improved the coverage of the level II axilla, and the IMN chain. The dosimetry benefits resulted in improving the therapeutic ratio, which included a minimized risk of treatment-related mortality. One of the most recent work concerns a comparison between IMPT and VMAT for regional nodal irradiation in breast carcinoma patients [41], where 20 patients were included in the analysis, 10 after breast-conserving surgery group and 10 post-mastectomy patients with tissue expander implants. Robust optimization methods were performed to evaluate the results. They also conducted a risk assessment of secondary cancer induction. For both groups of patients, proton therapy was a promising approach, especially when nodal volumes were irradiated. The dosimetry dose distribution from all studies is included in Table 2.

### 3.2. Skin Toxicity

In clinical practice, skin toxicity is assessed based on the criteria of the Common Terminology Criteria for Adverse Events (CTCAE) version 4.0 [49]. Verma et al. [11] investigated a study that assessed acute toxicity outcomes in breast cancer patients treated with adjuvant proton beam therapy. A group of 91 patients was examined. Skin symptoms were observed weekly during treatment, one month after treatment, and then 6 months after the end of radiotherapy. The observations lasted 15.5 months. It was difficult to evaluate and interpret patients’ relationships or classifications because the examined women were highly heterogeneous, with a combination therapies and tumor characteristics. Scientists [38] also investigated proton therapy’s clinical effects in locally advanced breast cancer treated with post-mastectomy proton radiation in a prospective clinical trial. This publication assessed mainly skin toxicity effects, determining the level of fatigue and radiation pneumonitis after 4 and 8 weeks of therapy. The patients’ maximum side effects appeared in the form of grade 2 skin toxicity, with the highest possible score being grade 3, as set by the CTCAE. They showed that using a proton beam was an appropriate method of treating breast tumors, with an acceptable toxicity, but it required further research. In 2015, another work was done to assess skin toxicity in a group of 30 patients treated with proton therapy [44]. The characteristics of the patients and the obtained dosimetry values were collected. Observing the effects weekly during treatment, one month after radiotherapy, and three to six months later, they concluded that postoperative proton therapy was well tolerated while achieving an acceptable level of skin toxicity comparable to photon therapy. The proton beam could be planned with a very high conformality. They also highlighted that this research needed to be developed further, especially in the field of cardiopulmonary toxicities. Tommasino et al. [47] focused on applying the skin normal tissue complication probability (NTCP) model to optimize the IMPT technique in treating left-sided breast cancer, due to skin toxicity and poor cosmesis after treatment. In this study, 10 patients who underwent proton beam irradiation and breast-conserving surgery were selected. New plans for proton radiation with and without skin optimization were calculated. Because of the skin NTCP model, the evaluated values presented a lower toxicity of applied radiotherapy in implementing skin optimization strategy. IMPT showed better values in heart and lung sparring. because of the model used, the risk of cardiac events after treatment was reduced. The group concluded that IMPT was a safer form of breast cancer irradiation without any noticeable increase in skin toxicity, introducing new optimizations into the treatment planning system. Postmastectomy proton radiotherapy was also studied, taking into account skin toxicity compared with photon radiotherapy [48]. In this study, 42 patients were irradiated with adjuvant chest wall and regional nodal proton therapy. The most common toxicity symptoms were dermatitis, fatigue, and esophagitis, but without noticeable grade 3 or 4. The follow-up analysis showed an excellent locoregional control rate of 96% as the longest follow-up known so far, but it is worth noting that longer follow-up and randomized clinical trials are needed. Liang et al. [50] conducted a study to identify the prognostic factors giving information on the possible occurrence of grade 3 radiation dermatitis in 43% of patients following passive-scattering proton therapy. They observed that the DVH of D10cm3 and V52.5 cobalt Gy equivalent to 5 mm skin parameters were related to a risk of skin toxicity, while finding a correlation with smoking. This study is a promising tool aimed at identifying high-risk patients for whom this form of treatment may be modified in order to avoid the occurrence of grade 3 skin toxicity as much as possible. Bush et al. [51] updated their previous report of a phase 2 trial on proton radiotherapy effects in APBI with early-stage breast cancer. A group of patients underwent a partial mastectomy with negative margins. Proton radiation was delivered at a dose of 40 Gy to the surgical bed in 10 fractions. The results were described as clinical assessments and annual mammograms to monitor toxicity, tumor recurrence, and cosmesis over 60 months. Grade 3 skin toxicity was shown to be absent. However, 7 cases of grade 1 telangiectasia were observed.

### 3.3. Potential Causes of the Toxicity

The magnitude of response after irradiation can be caused by an extensive range of reasons. The most common are physical and biological causes. The physical factors include all of the inconveniences related to technical issues like planning robustness, imaging, geometrical uncertainties, plan optimization, motion management, tracking, gating, treatment monitoring, or implemented treatment patterns. These issues take place immediately before or during the therapy session. The biological part of the factors is usually directly related to the physiology and anatomy of the patient. After irradiation, the tumor response depends on molecular factors and the microenvironment, such as hormone-dependence, radiosensitivity, metastatic capacity, and resistance to a given type of treatment.

#### 3.3.1. Respiratory Motion

The location of the planned treatment volume may change during a treatment session because of the patient’s functional movements, so these shifts must be included in the dimension of the radiation beam [52]. The larger the radiation beam, the larger the area of healthy tissues surrounding the PTV that receives a high radiation dose [53]. For this purpose, irradiated volume position as a function of time should be defined [54,55].

To minimize the doses that may affect irradiation-related heart diseases, especially in left-sided tumors, a new technique called deep inspiration breath holding was proposed [56]. Respiratory gating (RG) is a concept that is similar to DIBH [57]. This method’s primary goal is to irradiate the tumor volume only during dedicated phases of the patient’s respiratory cycle, keeping the tumor as far away from the OAR as possible [19,58]. This technique is also used during proton beam irradiation. Oden et al. [46] examined the effect of breath motion on dose values in the heart, LAD, left lung, and target coverage. In their study, 12 patients showed regular breathing patterns. To precisely contour the breasts, they performed CT scans in the following three states: free-breathing (FB), breath-hold-at-inhalation (BHI), and breath-hold-at-exhalation (BHE). They showed that respiratory movements during proton beam irradiation had little effect on the quality of treatment plans compared with photon beam planning. The OAR dose results were not statistically different in terms of respiratory movements. However, they noted that the effect of the applied relative biological effectiveness (RBE) value during planning significantly influenced the dose levels by comparing the constant characteristic value for protons with the variable RBE with a greater practical value.

Implementing proton therapy is associated with high costs and technical difficulties [59]. For this reason, respiratory gating is typically not used during planning proton beam irradiation. Employment gating in the case of protons would increase the cost and time of the irradiation of the patient, which is undesirable [60]. A longer irradiation time could further disturb the irradiation course and cause more significant shifts in the irradiated area. Considering the pencil beam scanning system used in proton therapy, a potential correlation between the active delivery of radiation and the movement of the irradiated target was observed [61]. Anatomy variation also has a significant impact on dose blurring and deformation [62]. The primary form of prevention from higher doses in OAR is using fiducials markers while tracking inside the tumor volume [63]. In proton therapy, implementing such a solution is a technical challenge and cannot be used in all moving tumors. Newhauser et al. [64] simulated different materials and the size of markers in various combinations during proton radiotherapy. Introducing gating in proton therapy would result in lower doses in OARs. It is essential to determine the exact target distance from healthy organs and information on proton therapy for mobile tumors in future studies. Another group of scientists [65] studied the efficiency of the gating method in spot-scanning proton therapy. They performed a simulation using lung cancer patient’s tumor trajectory data. They aimed to investigate the reason for dose distribution deterioration with respect to tumor motion and gating. They concluded that in shallow-seated tumors, the Bragg peak’s accuracy in treatment plans is limited, mainly because of the range of target motions involved. This could be avoided by using an applicable number of beams and by placing an absorber of an appropriate thickness in the path of the beam at a depth greater than 12 cm. By utilizing plans generated for real patients, this work makes a significant contribution to the possibility of improving radiation dose distribution for moving tumors. Mast et al. [43] performed an analysis of the doses measured in the OAR during IMPT irradiation, comparing two treatment plans of 20 patients with left-sided breast tumor using two techniques—free-breathing and breath-holding. They aimed to estimate the most efficient method to achieve the lowest possible cardiotoxicity of radiotherapy affecting the heart and LAD. They compared the results achieved with IMRT and IMPT, observing a significant difference in values, favoring proton beam therapy.

In most cases, IMPT led to almost zero doses in OAR, although the difference in the respiratory technique during irradiation did not significantly reduce cardiotoxicity. This result was significant in practice and affected the patient’s comfort during the therapeutic session. The authors suggested that IMPT, although it was not the most accessible method, should be dedicated to patients with increased risk of cardiac events.

Patel et al. [45] conducted a study based on an analysis of the dosimetry results obtained after photon and proton radiation, comparing the use of two free-breathing techniques versus deep inspiration breath-hold. They included patients with post-mastectomy left-sided breast cancer and unfavorable cardiac anatomy. Moreover, they reached the following irradiation patterns: partially comprehensive tangent photon with DIBH, passively scattered proton during FB, pencil-beam scanning proton during FB, and PBS proton with DIBH. All of the plans generated for the analysis achieved the target’s expected coverage, including chest wall and regional lymphatics of 95%. Scientists showed that the breathing techniques used did not affect the dose values in the OAR when using a proton beam. However, the dose homogeneity and cardiopulmonary sparing were improved. As a result, it can be concluded that proton radiotherapy could be recommended for patients who find it challenging to adapt to the DIBH technique during irradiation, which is of great importance in everyday practice and for patients’ comfort in life.

#### 3.3.2. A Biological and Technical Issue

Over the years, advanced forms of proton radiotherapy techniques have been observed, ranging from the standard passive scanning method, to uniform scanning, to the modern pencil beam scanning technics.

Wilkens et al. [66] examined the potential clinical contribution of the differentiated RBE value to the IMPT scanning technique, as well as and the ways of implementing RBE into the inverse planning process. The RBE distribution was represented by models, described as a function of dose, LET, and tissue type. They prepared a research version of the software to take into account biological optimization using the example of prostate treatment plans, which allowed for direct optimization towards determining the physical dose. Biological optimization was compared with conventional physical dose optimization for IMPT by analyzing the distal edge tracking (DET) and full 3D modulation of beam spots. The 3D modulation showed a relatively homogeneous LET value in the PTV for the DET area, while at the boundaries of the irradiated area, there were much higher LET values. It should be noted that deviations from the fixed RBE value could be potentially dangerous when, for example, the critical organ is close to the Bragg peak. They introduced tools to estimate the effects of RBE on a given treatment plan to detect potentially disadvantageous situations, e.g., regions of elevated or depleted LET. They showed that the side effects of RBE introduction could be compensated by optimizations; however, obtaining a heterogeneous distribution in PTV was not functional to observe a relevant biological outcome. It can be concluded that this approach is a potential solution to the inconvenience of treatment planning.

It is commonly suggested and accepted that the RBE value in the dose distribution per unit depth of proton radiation penetration is not constant [67], in particular when analyzing the end of the particle path range. By implementing this information into daily practice, it can be concluded that the biologically effective dose distribution may be different from that planned in the system. Unfortunately, there are uncertainties and inaccuracies in the variable RBE models, as there is no clinically significant evidence of harmfulness when using a fixed RBE of 1.1. There is a need to improve the models predicting the actual RBE values in order to increase proton therapy’s therapeutic ratio in the future. Models predicting the biological effects can provide significant value to the estimation of clinical effectiveness, dose size, and predictable assumptions and scenarios based on Monte Carlo simulations.

Paganetti et al. [68] focused on comparing three controversial aspects by comparing the proton and photon radiation’s biological effectiveness. They considered clinical practice for a 10% change in efficacy over the prescribed dose, an approach to constructing proton dose distribution models from photon radiation, and estimating the risk of inducing second cancer by the presence of scattered radiation during proton beam irradiation. When analyzing RBE variations’ quantification, the values in in vivo and in vitro experiments differed significantly, ranging from 0.9 to 2.1 [69]. They also confirmed in their work that there is no significant and clear evidence not to use the RBE 1.1 value for protons, the only reason to consider making changes is when the target is close to the OAR. Grassberger et al. [70] generated plans for different α/β ratio values, and significant differences in the potential dose distribution changes were observed. It can be theorized that omitting the tissue parameter causes underestimating the RBE and, consequently, a change in distribution. Unfortunately, the models are not sufficient, and in vivo experimental studies are needed in order to confirm the dose distribution differences.

## 4. Future Perspectives

Choi et al. [71] tested the identification of genetic factors that may be correlated with the radio-sensitivity of breast cancer. Moreover, they analyzed the RBE of 230 MeV protons versus 6 MV X-rays, taking into account 10 breast cancer lines, including five designated as triple-negative breast cancer cell lines. They showed that the effects obtained for one cell line differed in value from the standard RBE. Furthermore, they demonstrated that the occurrence of cyclin D1 was correlated with the proton irradiation. The decreasing value of cyclin D1 increased the RBE proton in the two TNBC cell lines MDA-MB-231 and Hs578T cells. Because of this work, it can be concluded that the cyclin D1-CDK4-RB1 pathway may be a potential target in the radiotherapeutic treatment of TNBC with the use of a proton beam. The authors assisted in the search for creating genome-based precision proton therapy. Bravata et al. [72] analyzed and compared the response of breast cancer cell lines to proton and electron radiation, taking into account molecular factors and gene expression. They used the tumorigenic MCF7 cells (estrogen receptor-positive breast cancer cells), MDA-MB-231 cells (triple negative breast cancer cells), and the non-tumorigenic MCF10A cells. The effects of the gene expression differences were investigated based on the microarray cDNA against the variable LET using the dose of 9 Gy. Moreover, they performed a Monte Carlo analysis to estimate the LET distribution in the target region to gain insight into the physical effects of gene expression regulation-induced differences. Scientists showed the activation of various signals and molecular networks. It was observed that the factors and pathways related to the immune control process were induced in MCF10A and MDA-MB-231 cell lines. Observed deregulations of the Capn8 and Kirrel2 genes following proton irradiation are related to the degradation of the cellular matrix and apoptosis pathway, and to the regulation of insulin secretion. They also evaluated the unique and common deregulated gene lists after electron and proton irradiation in MCF10A, MCF7, and MDA-MB-231 cells. They identified 81 gene signatures of shared deregulated genes in both irradiation beams in MCF10A cells. The pathways of the selected gene signatures were connected through androgen, mineralocorticoid, and glucocorticoid biosynthesis; the regulation of IFNA signaling; and interleukin 7 signaling. The MCF7 cell line shared a deregulated 141 gene-signature in both kinds of irradiation. These gene-signatures were associated with different pathways, like transcriptional regulation by TP53, p53 responsive genes, and cell cycle inhibitor p21. Finally, the group observed that MDA-MB-231 cells deregulated 154 gene signatures after both irradiation modalities. The relevant pathways of the selected 154 gene signatures were related to histone acetyltransferases acetylate histones or RUNX1 regulate transcriptions of the genes involved in B cell receptor signaling. They concluded that the lines reacted similarly to ionizing radiation, no matter what type of beam was used.

Attempts were also made to look for cardiotoxicity indicators by analyzing the post-irradiation effects of the kininogen-deficient Brown Norway Katholiek (BN/Ka) rat model [73]. The volume of cardiac inflammation after local irradiation influenced by the Kallikrein–Kinin System (KKS) was tested. Rats were irradiated with a single dose of 18 or 24 Gy I, and again after 3 and 6 months. The authors focused on the observation of KKS and concluded that it plays a pivotal role in the effects of ionizing radiation-induced cardiotoxicity and the onset of developing inflammation. KKS likely changes the signaling of Erk½. This work suggests that pharmacological modifications of KKS may be a potential advantage for certain aspects of diseases caused by ionizing radiation.

The faster and more effective model-based approach was proposed to select patients who would benefit the most therapeutically from proton therapy [74]. The process consisted of two parts. The first concerned patient selection and the second considered the clinical validation of proton therapy with so-called sequential prospective observational cohort studies. Patients were selected according to the unfavorable dose distribution criteria and taking into account the specific parameters established in the treatment plans. The next scientific group [75] investigated a mechanism-based approach to predict the RBE effectiveness for the proton beam using the repair–misrepair–fixation (RMF) model. They also used the Monte Carlo Damage Simulation (MCDS) software to assess the radiation-induced effects like DNA double-strand breaks (DSB). They analyzed that doses in the range of 0.5 to 10 Gy corresponded to proton RBE values between 1.02 to 1.4. The observations allowed for the conclusion that the proposed approach constitutes a quantitative evaluation of the effects of particle LET on DSB induction. In correlation to the obtained results, predicting hot and cold spots within the SOBP in proton therapy is possible. They showed that the correlation between RBE for protons and tissue parameters was less critical. Moreover, the MCDS algorithm also made it possible to check the effect of oxygen correlated with DSB as a biological modelling approach.

Many more attempts are needed to help clinicians understand the molecular specification of data for a given cancer type in order to design cancer therapies more effectively. Moreover, understanding the response of cells by molecular type to different irradiation patterns showing gene expression changes should be the prime concern for research groups aiming to design more effective targeted therapy strategies [69,76].

An international expert workshop was conducted to update about recent studies about proton therapy’s radiobiology [22]. They emphasized that the potential of proton radiotherapy is not sufficiently exploited because of the wide range of limitations and the lack of precise knowledge of the issues involved. There is still a lack of patient stratification based on biomarker expression to determine patients with the highest likelihood profit from proton radiotherapy. Further research should focus on experiments with in vitro 3D cultures using late toxicity endpoints; systematic radiobiological studies analyzing repair, transduction signals, and anti-vascular effects and aspects related to chemotherapy; and targeted therapy in combination with protons.

Furthermore, it is essential to develop research on markers that can predict late toxicity after irradiation. There should be more emphasis on building databases. Prospective clinical trials are based on high-quality imaging, monitoring, collecting a comprehensive collection of biomaterials, and complete data from treatment planning systems. The creation of high-quality dosimetry data sets and outcomes to generate comparable data may impact the optimization of further research, hypotheses, clinical trials, and modelling systems for the quality of treatment with proton radiotherapy.

Another challenge is to create a database structured in terms of techniques, the same dose distribution parameters, and the patient’s factors. It is necessary to compare clinical data generated based on years of experience and follow-up, but unfortunately, such assumptions are difficult to implement. It does not change the fact that it should be done by modifying and improving the treatment planning systems and qualifying patients for the best-suited irradiation pattern in terms of technique and individual predispositions, as well as the molecular factors of the patient. Overall, many centers have compared the dosimetry results between proton and photon therapy. This is often done by producing a new treatment plan using scans of patients previously treated with a photon beam. On this basis, it is possible to compare the doses in the system using algorithms characteristic for a given type of radiation [77]. This is not a fully practical approach, because the dosimetry verification of these newly generated plans is also needed to ensure that the planned treatment meets all of the necessary therapeutic apparatus’s necessary conditions and capabilities. There is still a lack of observations and analyses of the treatment that may result in diseases related to the heart or circulatory system, or studies that assess the risk of such complications more accurately.

Unfortunately, the knowledge of the influence of respiratory movements on proton therapy quality requires further research, especially in complicated cases when the tumor is located deeply and in situations where there is a high risk of radiation-related cardiac diseases. However, this is an arguable point because of the high costs of implementing new solutions in proton facilities and the knowledge gap in the real usefulness of potential changes leading to lower cardiotoxicity. The above studies suggest that the toxicity of the proton beam has a more significant correlation with the currently used irradiation technique than with the occurrence of respiratory movements during the treatment session.

## 5. Conclusions

Since proton radiation has been proposed in cancer treatment, it has become a potential solution to reducing the cardiotoxicity of conventionally used photon radiation. Taking into account the analyzed aspects of minimizing the toxicity of proton therapy in breast cancer, we conclude that the use of a proton beam is an appropriate approach in the treatment of difficult-to-reach tumor locations. Studies showed a significant reduction in side effects related to cardiac risk events with the use of proton therapy.

Because of the favorable dose–depth distribution in proton radiation, it is possible to significantly minimize OAR doses [78]. Typically, dosimetry analysis, aimed at comparing the OAR dose values, relies on generating new proton treatment plans based on the previous ones planned for the photon beam. It is crucial to start comparing the real dosimetry doses and to design a new methodology in order to check the dosimetry values when carrying out radiotherapy for protons and photons [79].

Another problematic issue is that 15 to 20 years is needed to generate useful information from randomized clinical trials, which is unrealistic, because radiation therapy is a rapidly evolving field. It will be impossible to observe or compare techniques because of changes in criteria and the emergence of new strategies or technological solutions. Therefore, the observed effects over the years and the conclusions drawn from them cannot be applied in the future. Nevertheless, more research is being done to make radiation therapy more personalized so as to reduce the magnitude of side effects as much as possible.

## Figures and Tables

**Table 1:** Dosimetry of dose distribution for accelerated partial breast irradiation, taking into account the parameters of mean dose, Dmax, V5, and V20 in OAR (heart and lungs).

First Author	Year	Number of Patients	Target	Total Dose [Gy]	Delivery Technique	OAR	Mean Dose	Dmax	V5	V20
Taghian [26]	2006	25	APBI	32	3DCPT	**Heart**	0	-	0.00%	0.00%
						**Lungs**	-	-	3.00%	1.00%
Kozak [27]	2006	24	APBI	32	3DCPT	**Heart**	0.1	-	0.00%	0.00%
						**Left Lung**	0.5	-	3.00%	1.00%
Toscas [28]	2010	14	APBI, deep-seated tumors	16	IMPT	**Heart**	0.01	-	0.00%	-
						**Left Lung**	0.2	-	V3:1.80%	V10:0.20%
						**Right Lung**	0.01	-	V3:0.00%	-
Wang [29]	2013	11	APBI	38.5	PSPB	**Heart**	-	3.2	-	-
						**Lungs**	0.61	-	3.50%	0.10%
Galland-Girodet [30]	2014	19	APBI	32	PSPB	**Heart**	0	3.8	0.40%	0%
						**Lungs**	0.5	20.4	3.10%	0.70%
Hansen [31]	2015	12	APBI, breast cancer treated with SAVI	34	PSPB	**Heart**	0.0	0.43%	-	-
						**Left Lung**	0.04	34.41%	-	-

OAR—organ at risk; Dmax—maximum dose; V5—the volume of organ receiving 5 Gy; V20—the volume of organ receiving 20 Gy; APBI—accelerated partial breast irradiation; 3DCPT—three-dimensional conformal proton therapy; IMPT—intensity modulated proton therapy; PSPB—passive scattering proton beam; SAVI—Strut-Adjusted Volume Implant.

**Table 2:** Dosimetry of the dose distribution for accelerated partial breast irradiation, taking into account the parameters of the mean dose, Dmax, V5, and V20 in OAR (the heart, LAD, and lungs).

First Author	Year	Number of Patients	Target	Total Dose [Gy]	Delivery Technique	OAR	Mean Dose	Dmax	V5	V20
Fogliata [32]	2002	5	whole breast, left-sided breast cancer	50	PBS	**Heart**	2.2	19.3	-	-
						**Lungs**	3.5	43.8	10.40%	-
Lomax [33]	2003	no patients, the analyses of plans	whole breast, internal mammary, supraclavicular, and axillary nodes	50	PBS	**Heart**	5.8	53.8	39.0	-
						**Lung**	12.6	-	-	V50:11.5
Ares [34]	2010	20	whole breast, left-sided breast cancer	50	PBS	**Heart**	1.0	-	2.0	V22.5:0.0
						**Left Lung**	7.0	-	0.0	17.0
						**Right Lung**	0.0	-	0.0	0.0
MacDonald [42]	2013	12	whole breast, left-sided breast cancer after mastectomy	50.4	3DCPT	**Heart**	0.44	-	-	0.01%
						**Lungs**	6	-	-	12.70%
Jimenez [35]	2013	5	whole breast, left-sided breast cancer, bilateral implants	50.4	IMPT	**Heart**	-	-	2.80%	0.40%
						**Left Lung**	-	-	14.90%	4.30%
						**Right Lung**	-	-	13.50%	4.10%
Mast [43]	2014	20	whole breast	42.56	IMPT	**BH**				
						**Heart**	0.1	0.3	0.1%	0.0%
						**LAD-region**	0.3	1.8	0.4%	0.0%
						**Left Lung**	1.5	23.6	7.1%	2.5%
						**FB**				
						**Heart**	0.2	1.2	0.5	0.1
						**LAD-region**	0.7	4.5	2.8	9.7
						**Left Lung**	1.6	27	7.7	2.8
Xu [36]	2014	10	whole breast, left-sided breast cancer	50	3DCPT	**Heart**	1	-	7%	0%
						**Left Lung**	5.5	-	50%	31%
						**Right Lung**	0.4	-	1%	0%
Flejmer [37]	2015	10	whole breast, breast cancer (5 left-sided and 5 right-sided) postoperative radiation treatment	50	IMPT	**Heart**	0.2	-	-	-
						**LAD-region**	1.4	-	-	-
						**Left Lung**	6.3	-	V10:25.8%	10.50%
						**Right Lung**	0	-	-	-
Fagundes [38]	2015	10	left-sided stage III breast cancer after mastectomy, the chest wall, axilla levels I to III, the supraclavicular and internal mammary nodes (IMN)	50.4	PBS	**Heart**	1.2	-	-	V25:1.2
						**LAD-region**	7	27.6	-	-
						**Left Lung**	-	-	41.30	0.28
						**Right Lung**	-	-	0.3	0.04
Cuaron [44]	2015	30	27 left-sided, 3 right-sided, nonmetastatic breast cancer, postoperative, unfavourable cardiopulmonary anatomy	50.4	PBS	**Heart**	1	-	5.00%	1.16%
						**Lungs**	-	-	34.35%	7.31%
Lin [39]	2015	10	whole breast, left-sided breast cancer	50	PBS	**Heart**	0.011	-	0.00%	0.00%
						**LAD-region**	0.031	-	-	-
						**Lungs**	0.88	-	4.70%	0.00%
Bradley [40]	2015	10	whole breast, BCT-breast-conserving therapy, postmastectomy	50.4	PBS	**Heart**	0.6	-	2.70%	1.00%
						**LAD-region**	1.7	30.5	-	-
						**Left Lung**	11.0	-	35.30%	21.60%
Patel [45]	2017	10	whole breast, left breast cancer referred for PMRT	50.4	PBS	**BH**				
						**Heart**	0.7	-	-	0.40%
						**LAD-region**	-	4.6	-	-
						**Left Lung**	7.5	-	-	14.43%
						**FB**				
						**Heart**	0.98	-	-	0.86%
						**LAD-region**	-	4.58	-	-
						**Left Lung**	7.49	-	-	14.43%
Oden [46]	2017	12	whole breast, left-sided breast cancer	50	IMPT	**Heart**	0.1	-	-	-
						**LAD-region**	1.6	-	-	-
						**Left Lung**	1.3	-	-	1.40%
Tommasino [47]	2017	10	whole breast, postoperative left-sided breast cancer, after conserving surgery	50	IMPT	**Heart**	0.5	-	-	0.60%
						**LAD-region**	0.7	-	-	-
						**Left Lung**	3.3	-	-	5.80%
Luo [48]	2018	42	whole breast, left-sided breast cancer after mastectomy	50.4	3DCPT	**Heart**	0.7	16.3	4.30%	0.50%
						**Left Lung**	-	-	34.00%	16.10%
De Rose [41]	2019	20	10 in the breast-conserving surgery group and 10 post-mastectomy patients	50	IMPT	**Heart**	0.4	-	-	-
						**Left Lung**	6.2	-	28.50%	12.20%

OAR—organ at risk; Dmax: maximum dose; V5—the volume of organ receiving 5 Gy; V20—the volume of organ receiving 20 Gy; APBI—accelerated partial breast irradiation; 3DCPT—three-dimensional conformal proton therapy; IMPT—intensity modulated proton therapy; PBS—pencil beam scanning; LAD—the left anterior descending artery; BH—breath holding, FB—free breathing.
